# CupAR negatively controls the key protein CupA in the carbon acquisition complex NDH–1MS in *Synechocystis* sp. PCC 6803

**DOI:** 10.1016/j.jbc.2024.107716

**Published:** 2024-08-22

**Authors:** Jiexi Liu, Fangfang Zheng, Min Xu, Teruo Ogawa, Hualing Mi

**Affiliations:** CAS Center for Excellence in Molecular Plant Sciences, Graduate Courses of the Chinese Academy of Sciences, Shanghai, PR China

**Keywords:** *Synechocystis* sp. PCC 6803, CO_2_-concentrating mechanism, NDH–1MS, CupA, CupAR

## Abstract

The low CO_2_-inducible NDH complex (NDH–1MS) plays a crucial role in the cyanobacterial CO_2_-concentrating mechanism. However, the components in this complex and the regulation mechanism are still not completely understood. Using a mutant only with NDH–1MS as active Ci sequestration system, we identified a functional gene *sll1736* named as *cupAR* (CupA Regulator). The *cupAR* deletion mutant, *ΔcupAR*, grew faster than the WT under high CO_2_ (HC) condition, more evidently at low pH. The activities of O_2_ evolution, CO_2_ uptake，NDH-1, and the building up of a transthylakoid proton were stimulated in this mutant under HC conditions. The *cupAR* gene is cotranscribed with the NDH–1S operon (*ndhF3-ndhD3-cupA*) and encoded protein, which specifically suppresses the transcription level of this operon under HC conditions. Mutation of *cupAR* significantly upregulated the accumulation of CupA, the key protein of NDH–1MS, under HC condition. CupAR interacted with NdhD3 and NdhF3, the membrane components of NDH–1MS, while accumulation of CupAR was reduced in the *ΔndhD3* mutant. Furthermore, CupAR was colocated with CupA in both NDH–1MS complex and NDH–1S subcomplex. On the other hand, deletion of *ndhR*, a negative regulator of the NDH–1S operon, increased the accumulation of CupAR, whereas deletion of *cupAR* significantly lowered NdhR. Based on these results, we concluded that CupAR is a novel subunit of NDH–1MS, negatively regulating the activities of CupA and CO_2_ uptake dependent on NDH–1MS by positive regulation of NdhR under enriched CO_2_ conditions.

Cyanobacteria possess a CO_2_-concentrating mechanism (CCM) that functions to elevate the CO_2_ concentration around the active site of ribulose-1,5-bisphosphate carboxylase/oxygenase to compensate for the low affinity of ribulose-1,5-bisphosphate carboxylase/oxygenase for CO_2_ (1). Two CO_2_-uptake systems and three HCO_3_^-^ transporters have been identified in *Synechocystis* PCC 6803 (hereafter *Syn* 6803) and other cyanobacterial strains (2). One of the CO_2_ uptake systems, NDH–1MS' complex consists of NdhD4, NdhF4, and CupB (ChyX), is a constitutive system showing low affinity for CO_2_; another NDH–1MS complex, consisting of NdhD3, NdhF3, and CupA (ChyY), is inducible under low CO_2_ (LC) conditions and has high affinity for CO_2_ (3, 4, 5). The expression of *ndhF3-ndhD3-cupA-cupS* operon was induced in *Syn* 6803 and *Synechococcus* PCC 7002 under LC conditions (6). The proteins encoded by *ndhF3-ndhD3-cupA-cupS* formed a small complex NDH–1S, in which CupA and a small protein CupS were identified as subunits of cyanobacterial NDH-1 (7, 8). Single-particle electron microscopy of purified NDH–1MS complex of *Thermosynechoccus elongates* revealed a U-shape structure (9). CupA is responsible for the U-shape by binding at the tip of the membrane-bound arm of NDH–1MS in *Thermosynechoccus elongatus* and *Syn* 6803 (10). Both CupA and CupB may have a CA-like activity (11, 12), similar to the thylakoid membrane–bound CA. EcaB regulates the conversion of CO_2_ to HCO_3_^-^ (13). The structure of NDH–1MS has also been resolved by cryo-EM (14). CupB has been purified and identified in a 450 kDa NDH–1MS' complex (15).

CCMs are upregulated under LC conditions, whereas downregulated under HC conditions (elevated CO_2_ levels) at the transcriptional level (16). Several transcriptional regulators participate in this process. CmpR as a regulator activates the expression of the *cmp*-operon encoding the high-affinity HCO_3_^-^ transporter BCT1 (17). NdhR (CcmR, encoded by *sll1594*) acts as a repressor of the genes involved in CCM, such as NDH–1MS and *sbtA*, under HC conditions (16, 18, 19). Some NdhR-regulated genes are inducible under LC conditions, suggesting that additional NdhR-independent Ci-regulatory mechanisms exist in cyanobacteria (20). Both NdhR and CmpR belong to a large family of LysR-type transcriptional regulators (21). 2-phosphoglycolate and RubP can enhance the promoter binding of CmpR (22) whereas 2-oxoglutarate and NADP^+^ function as corepressor for NdhR (23). Jiang *et al.* showed that the full-length structure of NdhR from *Syn* 6803 forms a complex with 2-oxoglutarate, and the NdhR regulatory domain interacts with 2-phosphoglycolate (24).

In this work, we identified a novel functional gene *sll1736*, named as *cupAR* (CupA Regulator) in *Syn* 6803, and found that *cupAR*-defective mutant, *ΔcupAR*, grew faster than the WT at HC conditions, especially under low pH, and showed higher activities of O_2_ evolution and CO_2_ uptake. The *cupAR* gene belongs to the same operon of NDH–1S, functioning as a negative regulator for NDH–1S genes that specifically suppresses the transcript levels of this operon under HC conditions. CupAR interacted with NdhD3 and NdhF3 and colocalized with CupA in both NDH–1MS complex and NDH–1S subcomplex. Deletion of *ndhR* increased the accumulation of CupAR, whereas deletion of *cupAR* significantly lowered NdhR. Our results suggest that CupAR is a novel subunit of NDH–1MS and negatively regulates the transcription of NDH–1S operon, the translation of *cupA*, and the activity of NDH–1MS for CO_2_ uptake by positive regulation of NdhR under HC conditions.

## Results

### Mutation of *cupAR* upregulates NDH–1S activity under HC conditions

To investigate the components of NDH–1MS, we isolated the complex from solubilized thylakoid membrane *cupA-His6* using Ni column. MS/MS analysis of the fraction eluted by 150 mM imidazole indicated the presence of CupA and CupAR (Table S2) in addition to many potential proteins copurified with CupA (Table S1). The result suggested that the CupAR protein might be associated with CupA in NDH–1S.

**Figure 1:**
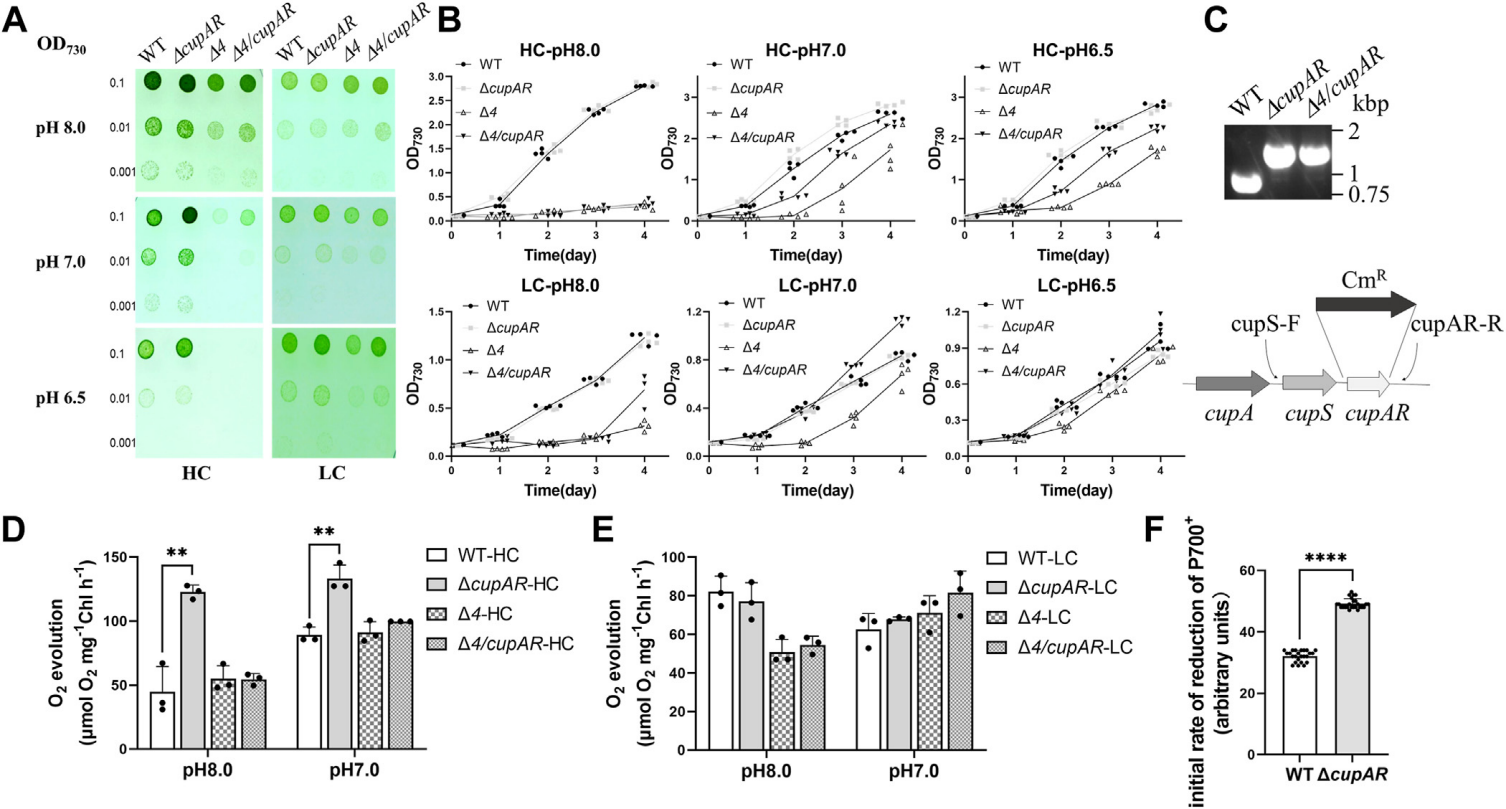
**Growth and photosynthetic properties of the WT and mutants.***A*, cells were grown on agar plates at pH 8.0, pH 7.0, and pH 6.5 under HC (10% CO_2_ [v/v] or LC [0.03%] conditions. *B*, growth curves of WT and mutants under different pHs and HC (10% CO_2_ [v/v] or LC [0.03%] conditions. *C*, The transposon insertion site and PCR amplification to confirm complete knockout of *cupAR* (*sll1736*) in the mutants; photosynthetic O_2_ evolution rate of WT and mutants under different pHs and HC (10% CO_2_ [v/v]) (*D*) or LC (0.03%) (*E*) conditions. *F*, the initial reduction rate of P700^+^ after far-red light. *Asterisk* indicates significant differences (*t* test, ∗*p* < 0.05, ∗∗*p* < 0.01, and ∗∗∗∗*p* < 0.0001). HC, high CO_2_; LC, low CO_2_.

To study whether CupAR participates in the function of NDH–1MS complex, we constructed *cupAR*-defective mutant by inserting Cm resistance cassette to *cupAR* gene (*sll1736*) in the WT strain. Complete segregation of *ΔcupAR* was confirmed by PCR analysis (Fig. 1*C*). In addition, we constructed *Δ4/cupAR* mutant by transforming the *Δ4* mutant (*ΔndhF4/ΔbicA/ΔcmpB/ΔsbtA*) with genomic DNA isolated from *ΔcupAR* mutant. Comparison of their growth with the WT on BG11 agar plate buffered at various pHs under HC (10% CO_2_) or LC (air CO_2_) indicated that the *ΔcupAR* and *Δ4/cupAR* mutants grew faster than the WT and *Δ4*, respectively, especially at low pH (below 8.0) under HC conditions (Fig. 1*A*, *left*), where the expression of genes in NDH–1S operon was suppressed and also under LC conditions at pH 6.5. In contrast, the difference became insignificant at pH 8.0 under LC conditions (Fig. 1*A*, right). A similar trend was found in the growth curves (Fig. 1*B*).

### Knockout of *cupAR* did not affect photosystem II activity but enhanced cyclic electron flow

No significant differences were observed between WT and *ΔcupAR* in the chlorophyll (Chl) fluorescence parameters, such as Q_A_, reflecting the redox state of electron transport carriers (Fig. S1*A*) and potential quantum efficiency of photosystem II (PSII), yield (II) as the indicator of the quantum yield of PSII (Fig. S1*B*). However, the cyclic electron flow around PSI was enhanced, as reflected by a transient increase in Chl fluorescence after termination of actinic light illumination (25) (Fig. S1*C*) and reflected by the initial reduction of P700^+^ after termination of far-red light (26) (Fig. 1*F*). The result indicates that CupAR negatively controls cyclic electron flow.

### Knockout of *cupAR* stimulates the activity of photosynthetic O_2_ evolution and CO_2_ uptake under HC conditions

The activity of photosynthetic oxygen evolution of *ΔcupAR* mutant was higher than that of the WT under HC (10% CO_2_) at pH 7.0 or pH 8.0 (Fig. 1*D*). There was no significant difference among WT, *Δ4*, and *Δ4/cupAR* either at pH 7.0 or pH 8.0 under HC conditions (Fig. 1*D*) and also between WT and *ΔcupAR*, or between *Δ4* and *Δ4/cupAR* at pH 7.0 or pH 8.0 under LC conditions (Fig. 1*E*). As shown in Fig. S1*D*, the rate of photosynthetic O_2_ evolution in *ΔcupAR* was higher than that in the WT by about 10 to 20% at pH 6.5 to 8.0 but was lower in the *Δ4* mutant by about 60%. However, the activity was partially recovered in *Δ4/cupAR* by about 6% at pH 8.0 and more evidently at pH 7.0 and 6.5. On the other hand, the rate of photosynthetic CO_2_ uptake in *ΔcupAR* increased by 24% at pH 8.0 and more significantly at lower pHs, by 43% at pH 7.0 and by 40% at pH 6.5, compared with that of WT. The CO_2_ uptake rate in *Δ4* was also decreased to about 26% of the WT at pH 8.0 and more obviously at lower pH. It was partially recovered in *Δ4/cupAR* by about 30% at pH 8.0, 16% at pH 7.0, and 12% at pH 6.5, respectively (Fig. S1*E*). The results suggest that CupAR stimulates the activity of CO_2_ uptake of NDH–1MS under HC conditions.

### Interaction between CupAR and NdhD3, NdhF3 *in vitro*

**Figure 2:**
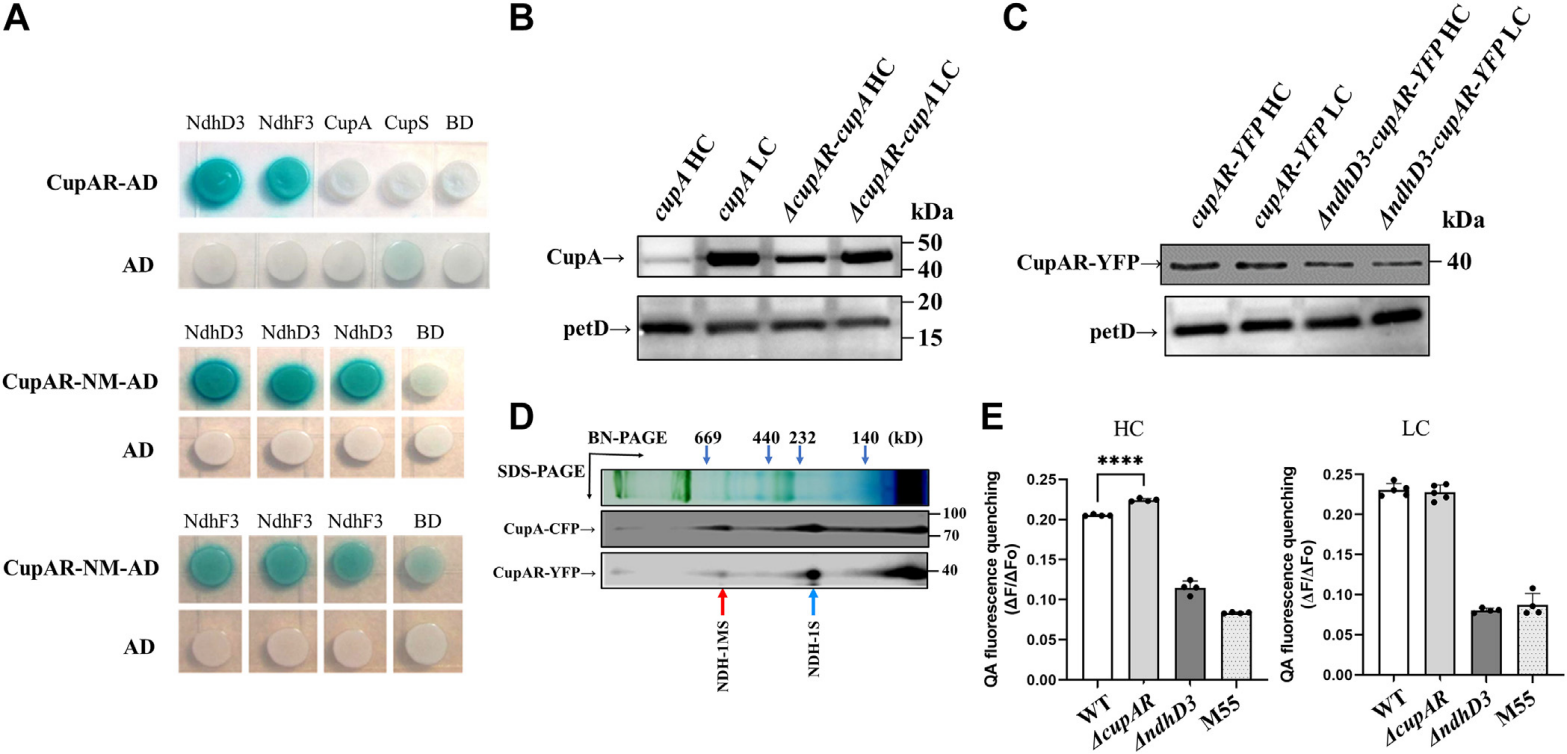
**Interaction, localization, and function of CupAR in NDH–1MS.***A*, yeast two-hybrid assay of CupAR interaction with NdhD3 and NdhF3. *Blue precipitate* represents accumulated β-galactosidase activity resulting from the activation of the lacZ reporter gene by protein–protein interaction. The induction plate was incubated at 30 °C for 28 h and then photographed. The interaction of AD with BD was assayed as a negative control. At least six independent experiments were performed, and one representative result is shown on each assay. *B*, Western analyses of CupA in the *ΔcupAR* mutant under LC and HC conditions. PetD, a subunit of cytochrome *b6f* complex, was used as control. *C*, the expression of CupAR in the *cupAR-YFP* and *ΔndhD3* strains. PetD was used as control. *D*, Western analyses of NDH–1MS and NDH–1S complexes after separation by 2D-gel electrophoresis (BN-PAGE and SDS-PAGE) of thylakoid membrane proteins. The strains were probed with specific antibodies against GFP. The positions of NDH–1MS and NDH–1S complexes, respectively, in WT are indicated by *red* and *blue arrows*. *E*, analysis of proton gradient across the thylakoid membranes using QA (quinacridine) fluorescence quenching in WT and mutants, *ΔcupAR*, *ΔndhD3*, and M55. Intact cells of WT and mutant strains were harvested at midlogarithmic phase (optical density at 730 nm ≈ 0.5) and then suspended at a final chlorophyll concentration of 10 μg ml^−1^ in fresh BG11 medium with 5 μM quinacridine. The quenching of QA fluorescence was induced by illumination with actinic light (60 μmol photos m^–2^ s^–1^) after starting measurement. The QA fluorescence quenching was calculated as the ratio (ΔF/ΔFo) of the decreased fluorescence intensity (ΔF) to the background fluorescence intensity (ΔFo). Values are means ± SE of three independent measurements. *Asterisk* indicates significant differences (*t* test, ∗*p* < 0.05, ∗∗*p* < 0.01, and ∗∗∗∗*p* < 0.0001). HC, high CO_2_; LC, low CO_2_; NDH, NAD(P)H dehydrogenase.

To confirm whether CupAR is a component of NDH–1S, we investigated the interaction between CupAR and the components of NDH–1S (NdhD3, NdhF3, CupA, and CupS) using a yeast two-hybrid system. As shown in Figure 2*A*, there was strong interaction of CupAR with NdhD3 and NdhF3, respectively. No interaction was found between CupAR and CupA or CupS *in vitro*. The *cupAR* (*sll1736*) encodes a protein of 127 amino acids with two transmembrane regions (amino acids 15–37 and 41–63) mainly on the N-terminal region and a hydrophilic tail of 64 amino acids on the C-terminal region, suggesting that the N terminus of CupAR may insert into the thylakoid membrane and interact with NdhD3 and NdhF3. To test this possibility, we synthesized CupAR-NM containing transmembrane region of CupAR (1–63 amino acids) as AD to detect the interaction with NdhD3 and NdhF3 as BD, respectively. The data showed that the interaction can still be detected between CupAR-NM and NdhD3 or NdhF3, indicating that the CupAR-NM is essential for its interaction with the NdhD3 and NdhF3.

### Effect of CupAR protein on other subunits in NDH–1S complex

As shown in Figure 2*B*, the expression of CupA was hardly detected under HC condition but was induced under LC condition in WT. The expression of CupA was greatly increased in the *ΔcupAR* mutant under HC condition. The expression level of CupAR was similar either under HC or LC conditions but evidently reduced in *ΔndhD3* mutant (Fig. 2*C*), indicating that NdhD3 is important for accumulation of CupAR. In contrast, CO_2_ concentrations had no effect on the PetD, a subunit of cytochrome *b6f* complex.

### CupAR is localized in NDH–1MS and NDH–1S complexes

To confirm whether CupAR is colocated with CupA in NDH–1MS complex *in vivo*, we constructed *cupAR-YFP* mutant by the method as described above. The thylakoid membrane from *cupA-CFP* and *cupAR-YFP* was subjected to Blue Native-PAGE to separate the NDH–1S complex, followed by second dimension SDS-PAGE to separate the subunits of these protein complexes, which were then immunoblotted using an anti-GFP antibody. As shown in Figure 2*D*, CupAR is colocated with CupA in both NDH–1MS and NDH–1S complexes.

### CupAR negatively controls the proton gradient across the thylakoid membranes under HC conditions

Proton gradient formation across the thylakoid membranes measured using quinacridine (QA) fluorescence quenching was faster in *ΔcupAR* than in WT grown under HC condition, but there was no significant difference between them when cells were grown under LC condition (Fig. 2*E*). In contrast, the QA fluorescence quenching was lowered in both *ΔndhD3* and M55 cells, as reported in the previous study (12). The result confirms that CupAR negatively controls the function of NDH–1MS under HC conditions.

### The *cupAR* gene is cotranscribed with the *ndhF3-ndhD3-cupA* operon and suppresses the expression of the other genes within this operon under HC conditions

**Figure 3:**
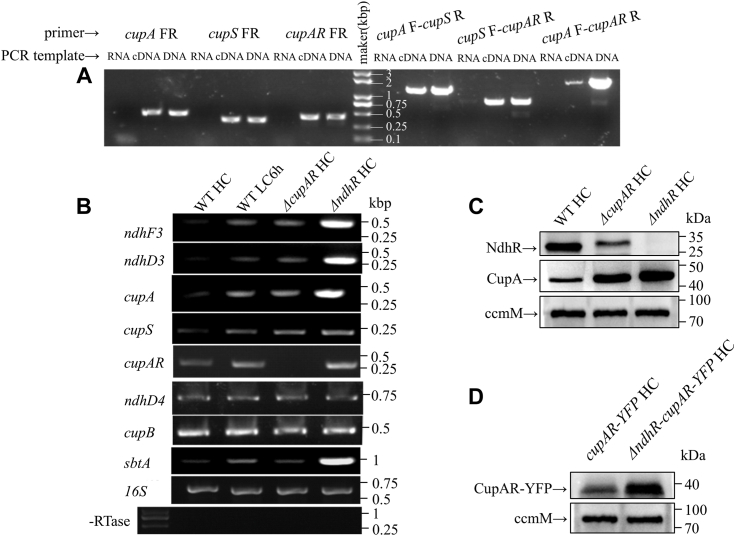
**Identification and analysis of the genes in *ndhF3-ndhD3-cupA* operon in transcriptional and translational levels.***A*, RT–PCR analysis of genes in *ndhF3-ndhD3-cupA* operon. *B*, expression of genes in *ndhF3-ndhD3-cupA* operon and *cupAR* under LC and HC conditions. 16S was used as control, and RTase was used as negative control. *C*, accumulation of NdhR and CupA in WT, *ΔcupAR* and *ΔndhR* observed by immunoblotting using antibodies against NdhR and CupA, respectively. CcmM, one of the components in carboxysome, was used as control. *D*, accumulation of CupAR in WT and *ΔndhR*. CcmM was used as control. CupAR in these strains was tagged with YFP and detected by immunoblotting using the antibody against YFP. Sample containing 20 μg thylakoid membrane proteins was loaded onto each lane. HC, high CO_2_; LC, low CO_2._

RT–PCR experiment showed that *cupS* and *cupAR* genes are cotranscribed with *cupA* (Fig. 3*A*), indicating that *sll1735*-*sll1736* belongs to the same operon. In HC-grown cells of WT, expression of genes in this operon except *cupAR* was hardly observed. The expression level of these genes was elevated after the cells were shifted to LC for 6 h. Surprisingly, in HC-grown cells of the *ΔcupAR* mutant, the expression levels of these genes were increased to those of LC-adapted WT cells (Fig. 3*B*). However, the degree of upregulation was much weaker as compared with that in the *ΔndhR* mutant. Furthermore, the expression of *sbtA* was not significantly increased in the HC-grown cells of the *ΔcupAR* mutant (Fig. 3*B*). As expected, the expression level of *ndhD4* and *cupB* of the constitutive CO_2_ uptake system kept unchanged under all conditions (Fig. 3*B*). The results indicate that CupAR specifically suppresses the transcription of the genes in NDH–1S.

### CupAR positively regulates the expression of NdhR and negatively regulates the expression of CupA

The aforementioned results suggest that CupAR might function as a negative regulative factor for expression of genes in the *ndhF3-ndhD3-cupA* operon. To understand the role of CupAR and NdhR for expression of genes in the operon, the accumulation of CupA was determined in the WT, *ΔcupAR*, and *ΔndhR* strains. Immunodetective analysis showed that NdhR level in *ΔcupAR* was about 30% of that in the WT (Fig. 3*C*). In contrast, the CupA level was upregulated in *ΔcupAR* and much more significantly in *ΔndhR* (Fig. 3*C*), indicating CupAR positively regulates NdhR, which negatively regulates the expression of genes in the *ndhF3-ndhD3-cupA* operon. On the other hand, the accumulation of CupAR was remarkably increased in *ΔndhR* (Fig. 3*D*), indicating that accumulation of CupAR is negatively controlled by NdhR.

## Discussion

Reverse genetics and proteomics revealed that the LC-inducible NDH–1MS is involved in CO_2_ uptake, which shows high affinity to CO_2_ (7). However, the components and activity regulation of this complex are still not completely understood. In this work, we identified a novel gene, *cupAR*, that negatively controlled the high affinity CO_2_ uptake system: the *ΔcupAR* mutant grew faster than the WT under HC conditions especially at pH below 8.0 (Fig. 1*A*). The recovery of growth of *Δ4* by further deletion of *cupAR* (*Δ4*/*cupAR*) supports the view that CupAR acts as a negative regulator for the NDH–1MS function (Fig. 1, *A* and *B*). In contrast to the genes involved in high-affinity CO_2_ uptake, such as *ndhD3*, *ndhF3*, and *cupA* (27), *cupAR* was constitutively expressed under HC conditions (Figs. 2*C*). Knocking out of *cupAR* gene stimulated accumulation of CupA (Fig. 2*B*) and expression of genes in *ndhF3-ndhD3-cupA* operons (Fig. 3*B*), which raised the growth, photosynthetic O_2_ evolution (Figs. 1*D* and S1*D*), and CO_2_ uptake (Fig. S1*E*) of the mutant under HC conditions.

*CupA, cupS*, and *cupAR* are expressed as an operon, possibly together with *ndhD3* and *ndhF3* (Fig. 3*A*). Moreover, mutation of *cupAR* remarkably increased the transcript level of genes in NDH–1S (Fig. 3*B*) and the accumulation of CupA (Fig. 3*C*) under HC conditions. The result suggests that CupAR negatively regulates the expression of genes encoding the subunits of NDH–1S, inactivation of *cupA* did not change the expression of *cupAR* (data not shown), while the amount of CupAR was decreased obviously in Δ*ndhD3* mutant (Fig. 2*C*). The increase of cyclic electron flow (Figs. 1*F* and S1*C*) and transthylakoid membrane proton gradient (Fig. 2*E*) by inactivation of *cupAR* indicate the negative regulation of NDH-1 by CupAR. Furthermore, yeast two-hybrid analysis showed that both NdhD3 and NdhF3 interact with CupAR *in vitro* (Fig. 2*A*), indicating that NdhD3 is crucial for accumulation of CupAR and might be the binding site for CupAR. In contrast to NdhR, CupAR only functions in negative regulation of the genes in the NDH–1S operon under HC conditions, and the suppressing effect is much weaker compared with that of NdhR (Fig. 3). The results suggest that CupAR specifically regulates the genes in NDH–1S and its activity under HC conditions.

**Figure 4:**
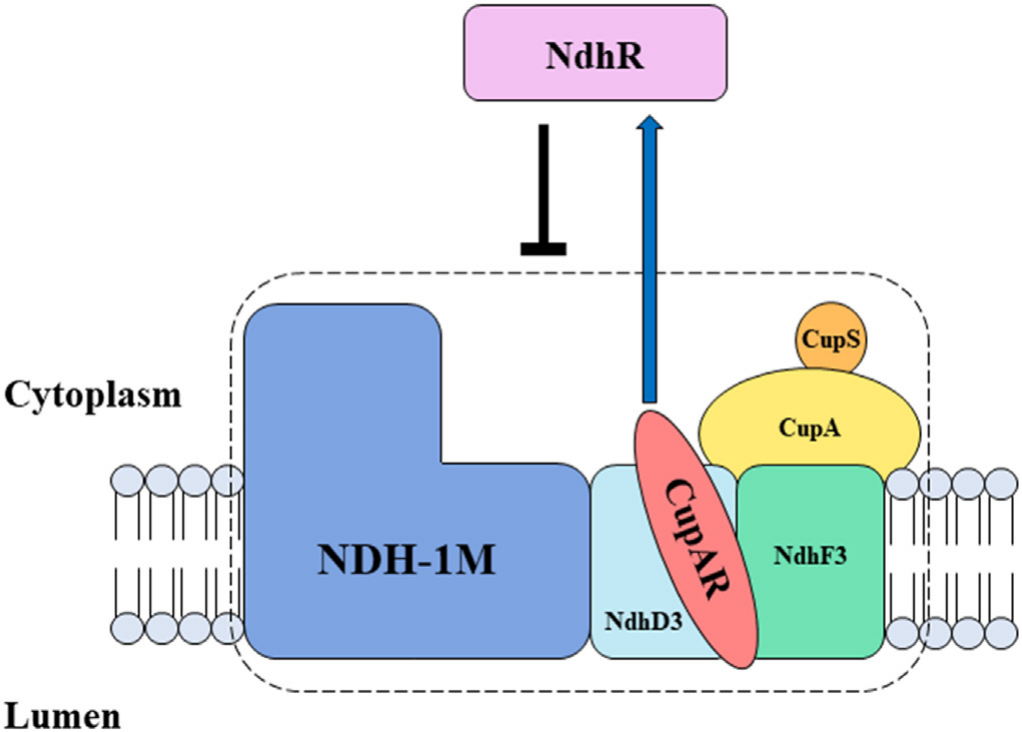
**Working mode of CupAR.** CupAR is located in NDH–1MS with its N terminus linked to NdhD3 and NdhF3. CupAR positively regulates NdhR, which negatively regulated NDH–1MS under HC conditions. HC, high CO_2_; NDH, NAD(P)H dehydrogenase.

CupAR is colocated with CupA in NDH–1MS and NDH–1S (Fig. 2*D*), and its N-terminal region interacts with NdhD3 and NdhF3 (Fig. 2*A*). Based on these results, we propose a model for localization of CupAR in NDH–1MS as depicted in Figure 4. The model also indicates that CupAR enhances the expression of NdhR (Fig. 3*C*), which suppresses the expression of genes in NDH–1MS (Fig. 3*B*) and its activity (Figs. 1*F*, 2*E*, and S1*C*) under HC or low pH conditions.

Thus, we may conclude that CupAR is a novel subunit of NDH–1MS, negatively regulating the activities of CupA and CO_2_ uptake dependent on NDH–1MS by positive regulation of NdhR. Our findings provide new insights into the regulation of inorganic carbon acquisition systems in cyanobacteria (1, 2).

## Experimental procedures

### Culture conditions

WT and mutant cells of *Syn* 6803 were grown in BG-11 medium buffered with 10 mM Tris–HCl, pH 8.0, at 30 °C under 50 μmol photons m^−2^ s^−1^ in the presence of 3% CO_2_ in air (v/v) to obtain HC cells. LC cells were obtained by aerating HC cells with air overnight under the same light conditions. BG-11 plates were prepared by adding 1.5% agar, 10 mM Tris–HCl (pH 8.0), and 0.3% Na_2_S_2_O_3_. Appropriate antibiotics were added into BG-11 medium for growing the mutants and complemented strains.

### Isolation and construction of mutants

The *cupA-His* mutant was made by adding 6-His to the C-terminal region of *cupA* gene. The *cupAR*-defective mutant, *ΔcupAR*, was made by replacing the *sll1736* gene with chloramphenicol cassette as shown in Figure 1*B*. The *Δ4* mutant (*ΔndhF4/ΔbicA/ΔcmpB/ΔsbtA*) of *Syn* 6803 possessing only the CupA-dependent CO_2_ uptake system was constructed and transformed with *ΔcupAR*-Cm construct to make *Δ4*/*cupAR* mutant. Transformation of *Syn* 6803 and *cupA-CFP* strain of Syn 6803 was constructed according to the method of Williams and Szalay (28). The construction and identification of other mutants, *ΔcupA*, *cupA-CFP*, and *cupAR-YFP-6His*, are shown in Figs. S2–S4, respectively. Primers used are listed in Table S3. The complete segregation of mutants was confirmed by PCR (Figs. S2–S4).

### Chlorophyll fluorescence measurement

Chl fluorescence was measured using a portable pulse amplitude–modulated fluorometer (PAM-2000) (Walz) (29). First, 1 ml suspension of *Syn* 6803 cells was pipetted into a reaction cuvette with a thermostat (30 °C). One end of a multibranched fiber optic bundle was inserted into the cuvette, and while the other ends were connected to the emitter–detector unit and to different light sources. The photochemical quenching coefficients (qP) and the effective yield of PS II photochemistry ([yield (II)]) were automatically calculated by the data acquisition software DA-2000 installed in a computer connected to PAM-2000. In the example given, the intensity of red actinic light increased stepwise every 20 s. At the end of each intensity step, the fluorescence ratio parameters, including yield (II), were assessed with the application of a saturation pulse. The kinetics of the fluorescence induction curve was recorded on a computer and then exported to Excel for graphical display of the light saturation curves. The postillumination increase in Chl fluorescence was monitored using a PAM Chl fluorometer (Walz) in an emitter–detector–cuvette assembly (ED-101US) and a 101ED unit, as previously described (25, 30, 31).

### Measurement of the redox state of P700

The redox change of P700 was monitored by absorbance at 810 to 830 nm, using a PAM Chl fluorometer (Walz), with an ED-P700DW-II unit for P700 absorbance changes as previously described (32). The cell suspension (1 ml) was added to a cuvette in the ED-101 US connected to far-red light with multibranched fiber optics (FR, *>*720 nm). Each sample was kept in the dark for 2 min to promote adaptation to dark conditions prior to measurement. Experiments were repeated three times.

### Oxygen evolution and CO_2_ uptake

The WT and mutant cells were harvested by centrifugation and resuspended in fresh BG11 with 10 mM NaHCO_3_ at 5 μg Chl *a* ml^−1^. Photosynthetic oxygen evolution was measured using a Clark-type oxygen electrode (Oxylab2; Hansatech) with a saturating light (150 μmol photons m^−2^ s^−1^) at 30 °C. Data represent the means ± standard deviations of values from three cultures in parallel. CO_2_ uptake was measured with the LI-6400/LI-6400XT Portable Photosynthesis System (LICOR). Cells were concentrated into 100 of optical density at 730 nm and then spread with 30 μl on an agar plate as a spot into a diameter of 0.5 cm incubated in the growth chamber before measurement. Each spot was cut out into a square of 1 cm × 1 cm and put on a cover glass before measurement. Same size of agar was used as control.

### Isolation of total membrane fraction

Five liters of 4-day-old cells (optical density at 730 nm = 0.6) were harvested, resuspended in 30 ml of buffer A (20 mM Hepes, pH 7.5, 5 mM sodium phosphate, 10 mM NaCl, 10 mM MgCl_2_, and 25% glycerol), and were broken by vortexing for 10 times at the highest speed for 20 s with 3 min interval cooling on ice. To remove the glass beads, the sample was centrifuged at 5000*g* for 10 min, and the membranes were subsequently collected by ultracentrifugation at 150,000*g* for 40 min and resuspended in buffer A.

### Electrophoresis and immunoblotting

Blue Native-PAGE for thylakoid membranes was performed as described earlier (30) with slight modifications. Thylakoid membranes were washed with 330 mM sorbitol, 50 mM Bis–Tris, pH 7.0, and 0.5 mM PMSF (Sigma) and resuspended in 20% glycerol (v/v), 25 mM Bis–Tris, pH 7.0, 10 mM MgCl_2_, 0.1 units of RNase-free DNase RQ1 (Promega) at a Chl *a* concentration of 0.6 mg ml^−1^. The samples were incubated on ice for 10 min, and an equal volume of 3% n-dodecyl-β-maltoside was added. The solubilization was performed for 1 h on ice. Insoluble components were removed by centrifugation at 18,000*g* for 15 min. The collected supernatant was mixed with one-tenth volume of sample buffer (5% Serva Blue G, 100 mM Bis–Tris, pH 7.0, 30% sucrose [w/v], 500 mM ε-amino-*n*-caproic acid, and 10 mM EDTA). Solubilized membranes were then applied to a 0.75-mm-thick, 5 to 13.5% acrylamide gradient gel (Mighty Small mini-vertical unit; Hoefer). Samples of 3 μg Chl *a* were loaded on the gel. Electrophoresis was performed at 4 °C by increasing the voltage gradually from 50 up to 200 V during the 5.5-h run.

For immunoblotting, the proteins were electrotransferred to a polyvinylidene difluoride membrane (Immobilon-P; Millipore) and detected by protein-specific antibodies using an ECL assay kit (Amersham Biosciences) according to the manufacturer’s protocol.

### RNA extraction and RT–PCR analysis

About 200 ml of *Syn* 6803 strains grown in BG-11 under air condition was collected by centrifugation and quickly frozen in liquid nitrogen. Total RNA was extracted using a TRIzol Reagent Kit (Invitrogen), according to the manufacturer’s instructions, and treated with RNase-free DNase I (TAKARA) for RT–PCR.

For RT–PCR, first-strand complementary DNA was synthesized in a 25 μl reaction mixture containing 50 mM Tris–HCl (pH 8.3), 75 mM KCl, 3 mM MgCl_2_, 10 mM DTT, 50 mM dNTPs, 25 U of RNasin, 200 U of M-MLV reverse transcriptase (Promega), 2 mg of total RNA, and 0.5 mg of random primers at 37 °C for 60 min. The relative concentration of complementary DNA was evaluated after serial dilutions by PCR using primers rnpB-F and rnpB-R and adjusted to the same level according to the brightness of PCR bands.

### Yeast two-hybridization assay

Yeast two-hybridization assay was performed using the LexA system (Clontech). The full-length *cupAR* and truncated *cupAR* gene (*cupAR-N*, *cupAR-M*, *cupAR-C*, *cupAR-NM*, and *cupAR-MC*) were cloned into the PJG4-5 vector to make the prey constructs. The fragments containing *ndhD3*, *ndhF3*, *cupA*, and *sll1735* were cloned into the PEG202 vector to make the bait constructs (primers are shown in Table S3). The bait and prey constructs together with a reporter vector pSH18-34 were cotransformed into the yeast strain EGY48 according to the manufacturer’s instructions for the Matchmaker LexA two-hybrid system (Clontech). Transformed yeast cells were diluted and dropped onto synthetic dropout plate containing X-gal and then were grown at 30 °C in darkness as described previously by Dai *et al.* (33).

### QA fluorescence quenching analysis

Fluorescence of QA was measured at 503 nm using the PAM chlorophyll fluorometer (Maxi-version; Walz) attached to a US-370 emitter with an emission peak at 375 nm and a PM-101/D detector as described previously (34, 35). Cells were harvested at logarithmic phase and suspended in reaction mixture of fresh BG11 medium with 5 μM QA at a final Chl concentration of 10 μg ml^–1^. The quenching of QA fluorescence was induced by illuminating the cells with actinic light (60 μmol photons m^–2^ s^–1^) after the background fluorescence became stable after about 2 min.

### Affinity chromatography

The membrane fractions of CupA-His were suspended with binding buffer at 1 mg Chl ml^–1^, solubilized with DM (1%) on ice for 1 h, and then centrifuged at 100,000*g* for 30 min. The supernatant of solubilized thylakoids was filtered through a 0.45-μm-pore-size membrane and applied on to the 1 ml Ni^2+^-affinity chromatography column. The column was washed with binding buffer containing 5 mM imidazole. Proteins were eluted with 3 ml of binding buffer containing 150 mM imidazole, and the eluted fractions were stored at −80 °C for further analysis.

### Identification of proteins by MALDI-TOF MS and electrospray ionization tandem MS

Proteins from eluted fractions of Ni^2+^-affinity chromatography column were digested with trypsin. The digests were analyzed by MALDI-TOF MS and electrospray ionization MS/MS as described by Battchikova *et al.* (36). The concentration of protein was determined using a detergent-compatible protein assay kit Bio-Rad.

### Bioinformatics tools

NCBI (National Center for Biotechnology Information), KEGG (Kyoto Encyclopedia of Genes and Genomes), and CyanoBase were used as sequence information resources. BLAST searches were performed to search for homologous sequences. To compare protein sequences or a protein sequence with an expressed sequence tag, FASTA and FASTX were applied, respectively. Domain analysis was performed by Pfam software (European Bioinformatics Institute).

## Data availability

Data can be shared upon request.

## Supporting information

This article contains supporting information.

## Conflict of interest

The authors declare that they have no conflicts of interest with the contents of this article.
