# Supplementary material for: CupAR negatively controls the key protein CupA in the carbon acquisition complex NDH–1MS in Synechocystis sp. PCC 6803
Source: J Biol Chem. 2024 Aug 22;300(9):107716. doi: 10.1016/j.jbc.2024.107716 (PMC11456788; doi:10.1016/j.jbc.2024.107716)
Supplement: Supporting information [file mmc1.docx]

#### Table S1. MS/MS identification of CupAR (sll1736) in the fraction eluted by 150 mM imidazole from NiNTA column loaded with solubilized thylakoid membrane of the with CupA-His6 tag.

| 1 | Elongation factor Tu OS=Synechocystis sp. (strain PCC 6803 / Kazusa) GN=*tuf* PE=1 SV=1 |
| --- | --- |
| 2 | DNA ligase OS=Synechocystis sp. PCC 6803 GN=*lig* PE=4 SV=1 |
| 3 | Uncharacterized protein OS=Synechocystis sp. PCC 6803 GN=BEST7613_2230 *sll1734*（*cupA*）PE=4 SV=1 |
| 4 | Cell division cycle protein OS=Synechocystis sp. PCC 6803 GN=BEST7613_5634 PE=4 SV=1 |
| 5 | 47 kD protein OS=Synechocystis sp. PCC 6803 GN=BEST7613_1343 PE=4 SV=1 |
| 6 | DNA helicase II OS=Synechocystis sp. PCC 6803 substr. PCC-N GN=*uvrD* PE=4 SV=1 |
| 7 | DNA-directed RNA polymerase subunit beta OS=Synechocystis sp. (strain PCC 6803 / Kazusa) GN=*rpoB* PE=3 SV=1 |
| 8 | Phycobiliprotein ApcE OS=Synechocystis sp. (strain PCC 6803 / Kazusa) GN=*apcE* PE=1 SV=1 |
| 9 | 1,4-alpha-glucan branching enzyme GlgB OS=Synechocystis sp. (strain PCC 6803 / Kazusa) GN=*glgB* PE=1 SV=1 |
| 10 | Probable glycogen synthase 2 OS=Synechocystis sp. (strain PCC 6803 / Kazusa) GN=*glgA2* PE=3 SV=1 |
| 11 | Ribulose bisphosphate carboxylase large chain OS=Synechocystis sp. (strain PCC 6803 / Kazusa) GN=*cbbL* PE=1 SV=1 |
| 12 | Carbon dioxide concentrating mechanism protein CcmM OS=Synechocystis sp. PCC 6803 substr. PCC-N GN=*ccmM* PE=4 SV=1 |
| 13 | Elongation factor G 2 OS=Synechocystis sp. (strain PCC 6803 / Kazusa) GN=*fusB* PE=3 SV=1 |
| 14 | Phosphorylase OS=Synechocystis sp. PCC 6803 substr. PCC-N GN=*glgP* PE=3 SV=1 |
| 15 | DNA-directed RNA polymerase subunit beta' OS=Synechocystis sp. (strain PCC 6803 / Kazusa) GN=*rpoC2* PE=3 SV=1 |
| 16 | ATP synthase subunit beta OS=Synechocystis sp. (strain PCC 6803 / Kazusa) GN=*atpD* PE=3 SV=1 |
| 17 | Sirohydrochlorin cobaltochelatase OS=Synechocystis sp. (strain PCC 6803 / Kazusa) GN=*cbiX* PE=1 SV=1 |
| 18 | Cyanophycin synthetase OS=Synechocystis sp. (strain PCC 6803 / Kazusa) GN=*cphA* PE=3 SV=1 |
| 19 | Uncharacterized protein OS=Synechocystis sp. PCC 6803 GN=BEST7613_5014 PE=4 SV=1 |
| 20 | Glyceraldehyde-3-phosphate dehydrogenase 2 OS=Synechocystis sp. (strain PCC 6803 / Kazusa) GN=*gap2* PE=1 SV=3 |
| 21 | Cell division protein FtsZ OS=Synechocystis sp. (strain PCC 6803 / Kazusa) GN=*ftsZ* PE=1 SV=1 |
| 22 | ATP synthase subunit alpha OS=Synechocystis sp. (strain PCC 6803 / Kazusa) GN=*atpA* PE=3 SV=1 |
| 23 | Ribonuclease D OS=Synechocystis sp. PCC 6803 GN=*rnd* PE=4 SV=1 |
| 24 | Uncharacterized protein OS=Synechocystis sp. PCC 6803 substr. PCC-N GN=*slr1302* PE=4 SV=1 |
| 25 | Allophycocyanin beta chain OS=Synechocystis sp. (strain PCC 6803 / Kazusa) GN=*apcB* PE=1 SV=1 |
| 26 | Putative methyl-accepting chemotaxis protein sll0041 OS=Synechocystis sp. (strain PCC 6803 / Kazusa) GN=*sll0041* PE=3 SV=2 |
| 27 | Molybdopterin biosynthesis MoeB protein OS=Synechocystis sp. PCC 6803 GN=*moeB* PE=4 SV=1 |
| 28 | C-phycocyanin alpha chain OS=Synechocystis sp. (strain PCC 6803 / Kazusa) GN=*cpcA* PE=1 SV=1 |
| 29 | UPF0272 protein slr1411 OS=Synechocystis sp. (strain PCC 6803 / Kazusa) GN=*slr1411* PE=3 SV=1 |
| 30 | Fructose-bisphosphate aldolase class 2 OS=Synechocystis sp. (strain PCC 6803 / Kazusa) GN=*fbaA* PE=1 SV=3 |
| 31 | DNA-directed RNA polymerase subunit gamma OS=Synechocystis sp. (strain PCC 6803 / Kazusa) GN=*rpoC1* PE=3 SV=1 |
| 32 | ATP-dependent Clp protease regulatory subunit OS=Synechocystis sp. PCC 6803 GN=*clpC* PE=3 SV=1 |
| 33 | 30S ribosomal protein S10 OS=Synechocystis sp. (strain PCC 6803 / Kazusa) GN=*rpsJ* PE=3 SV=1 |
| 34 | Acyl-[acyl-carrier-protein]--UDP-N-acetylglucosamine O-acyltransferase OS=Synechocystis sp. (strain PCC 6803 / Kazusa) GN=*lpxA* PE=3 SV=2 |
| 35 | Glutamine--fructose-6-phosphate aminotransferase [isomerizing] OS=Synechocystis sp. (strain PCC 6803 / Kazusa) GN=*glmS* PE=3 SV=3 |
| 36 | Uncharacterized protein OS=Synechocystis sp. PCC 6803 substr. PCC-N GN=*sll1884* PE=4 SV=1 |
| 37 | Chaperone protein ClpB 2 OS=Synechocystis sp. (strain PCC 6803 / Kazusa) GN=*clpB2* PE=3 SV=1 |
| 38 | Elongation factor Ts OS=Synechocystis sp. (strain PCC 6803 / Kazusa) GN=*tsf* PE=1 SV=3 |
| 39 | Uncharacterized protein OS=Synechocystis sp. PCC 6803 GN=BEST7613_1479 PE=4 SV=1 |
| 40 | Putative serine protease HtrA OS=Synechocystis sp. (strain PCC 6803 / Kazusa) GN=*htrA* PE=1 SV=1 |
| 41 | Pyruvate dehydrogenase OS=Synechocystis sp. PCC 6803 GN=BEST7613_5746 PE=4 SV=1 |
| 42 | Adenine phosphoribosyltransferase OS=Synechocystis sp. (strain PCC 6803 / Kazusa) GN=*apt* PE=3 SV=1 |
| 43 | GTP-dependent nucleic acid-binding protein EngD OS=Synechocystis sp. PCC 6803 GN=*ychF* PE=4 SV=1 |
| 44 | Uncharacterized protein OS=Synechocystis sp. PCC 6803 substr. PCC-N GN=*sll0572* PE=4 SV=1 |
| 45 | Uncharacterized protein OS=Synechocystis sp. PCC 6803 GN=BEST7613_4565 PE=4 SV=1 |
| 46 | Uncharacterized protein OS=Synechocystis sp. PCC 6803 GN=BEST7613_5930 PE=3 SV=1 |
| 47 | Uncharacterized protein slr1128 OS=Synechocystis sp. (strain PCC 6803 / Kazusa) GN=*slr1128* PE=3 SV=1 |
| 48 | Uncharacterized protein OS=Synechocystis sp. PCC 6803 substr. PCC-N GN=*sll1033* PE=4 SV=1 |
| 49 | Circadian clock protein kinase KaiC OS=Synechocystis sp. (strain PCC 6803 / Kazusa) GN=*kaiC* PE=3 SV=1 |
| 50 | Septum site-determining protein MinD OS=Synechocystis sp. (strain PCC 6803 / Kazusa) GN=*minD* PE=3 SV=1 |
| 51 | DNA-directed RNA polymerase subunit alpha OS=Synechocystis sp. (strain PCC 6803 / Kazusa) GN=*rpoA* PE=3 SV=1 |
| 52 | 30S ribosomal protein S1 homolog A OS=Synechocystis sp. (strain PCC 6803 / Kazusa) GN=*rps1A* PE=3 SV=1 |
| 53 | 30S ribosomal protein S3 OS=Synechocystis sp. (strain PCC 6803 / Kazusa) GN=*rpsC* PE=1 SV=3 |
| 54 | 30S ribosomal protein S1 homolog B OS=Synechocystis sp. (strain PCC 6803 / Kazusa) GN=*rps1b* PE=3 SV=1 |
| 55 | Ferric uptake regulation protein OS=Synechocystis sp. (strain PCC 6803 / Kazusa) GN=*fur* PE=3 SV=1 |
| 56 | Formyltetrahydrofolate deformylase OS=Synechocystis sp. (strain PCC 6803 / Kazusa) GN=*purU* PE=3 SV=1 |
| 57 | Uncharacterized protein OS=Synechocystis sp. PCC 6803 GN=BEST7613_4939 PE=4 SV=1 |
| 58 | Probable hydrogenase nickel incorporation protein HypB OS=Synechocystis sp. (strain PCC 6803 / Kazusa) GN=*hypB* PE=3 SV=1 |
| 59 | Mercuric reductase OS=Synechocystis sp. PCC 6803 GN=*merA* PE=3 SV=1 |
| 60 | High-affinity branched-chain amino acid transport ATP-binding protein OS=Synechocystis sp. PCC 6803 GN=*livG* PE=4 SV=1 |
| 61 | 1-deoxy-D-xylulose-5-phosphate synthase OS=Synechocystis sp. (strain PCC 6803 / Kazusa) GN=*dxs* PE=3 SV=1 |
| 62 | ATP synthase subunit beta OS=Synechocystis sp. PCC 6803 GN=*atpD* PE=3 SV=1 |
| 63 | GMP synthase [glutamine-hydrolyzing] OS=Synechocystis sp. (strain PCC 6803 / Kazusa) GN=*guaA* PE=3 SV=1 |
| 64 | Uncharacterized protein OS=Synechocystis sp. PCC 6803 GN=BEST7613_4593 PE=4 SV=1 |
| 65 | N5-carboxyaminoimidazole ribonucleotide synthase OS=Synechocystis sp. (strain PCC 6803 / Kazusa) GN=*purK* PE=3 SV=1 |
| 66 | Urease subunit alpha OS=Synechocystis sp. (strain PCC 6803 / Kazusa) GN=*ureC* PE=3 SV=1 |
| 67 | Uncharacterized protein OS=Synechocystis sp. PCC 6803 GN=BEST7613_2228 *sll1736*（*CupAR*） PE=4 SV=1 |
| 68 | Uncharacterized protein OS=Synechocystis sp. PCC 6803 substr. PCC-N GN=*sll0359* PE=4 SV=1 |
| 69 | Uncharacterized protein OS=Synechocystis sp. PCC 6803 substr. PCC-N GN=*sll0176* PE=4 SV=1 |
| 70 | C-phycocyanin beta chain OS=Synechocystis sp. (strain PCC 6803 / Kazusa) GN=*cpcB* PE=1 SV=2 |
| 71 | Ribulose bisphosphate carboxylase small chain OS=Synechocystis sp. (strain PCC 6803 / Kazusa) GN=*cbbS* PE=3 SV=1 |
| 72 | Universal stress protein Sll1388 OS=Synechocystis sp. (strain PCC 6803 / Kazusa) GN=*sll1388* PE=3 SV=1 |
| 73 | 50S ribosomal protein L7/L12 OS=Synechocystis sp. (strain PCC 6803 / Kazusa) GN=*rplL* PE=1 SV=3 |
| 74 | 50S ribosomal protein L10 OS=Synechocystis sp. (strain PCC 6803 / Kazusa) GN=*rplJ* PE=3 SV=1 |
| 75 | Urease accessory protein E OS=Synechocystis sp. PCC 6803 GN=*ureE* PE=3 SV=1 |
| 76 | N-acetyl-gamma-glutamyl-phosphate reductase OS=Synechocystis sp. (strain PCC 6803 / Kazusa) GN=*argC* PE=3 SV=1 |
| 77 | Phycobilisome 32.1 kDa linker polypeptide, phycocyanin-associated, rod 1 OS=Synechocystis sp. (strain PCC 6803 / Kazusa) GN=*cpcC1* PE=1 SV=3 |
| 78 | Ferredoxin--NADP reductase OS=Synechocystis sp. (strain PCC 6803 / Kazusa) GN=*petH* PE=1 SV=2 |
| 79 | Urea transport system ATP-binding protein OS=Synechocystis sp. PCC 6803 substr. PCC-N GN=*urtE* PE=4 SV=1 |
| 80 | Hybrid sensory kinase OS=Synechocystis sp. PCC 6803 GN=BEST7613_5969 PE=4 SV=1 |
| 81 | Cell wall-associated protein OS=Synechocystis sp. PCC 6803 GN=*wapA* PE=4 SV=1 |
| 82 | Tryptophan synthase alpha chain OS=Synechocystis sp. (strain PCC 6803 / Kazusa) GN=*trpA* PE=3 SV=1 |
| 83 | Type 2 NADH dehydrogenase OS=Synechocystis sp. PCC 6803 substr. PCC-N GN=*ndb* PE=4 SV=1 |
| 84 | 50S ribosomal protein L11 OS=Synechocystis sp. (strain PCC 6803 / Kazusa) GN=*rplK* PE=3 SV=1 |
| 85 | Epimerase family protein slr1223 OS=Synechocystis sp. (strain PCC 6803 / Kazusa) GN=*slr1223* PE=3 SV=2 |
| 86 | CheA like protein OS=Synechocystis sp. PCC 6803 GN=BEST7613_5708 PE=4 SV=1 |
| 87 | Acetolactate synthase small subunit OS=Synechocystis sp. (strain PCC 6803 / Kazusa) GN=*ilvH* PE=3 SV=2 |
| 88 | Uncharacterized protein OS=Synechocystis sp. PCC 6803 substr. PCC-N GN=*slr1104* PE=4 SV=1 |
| 89 | Uncharacterized protein OS=Synechocystis sp. PCC 6803 substr. PCC-N GN=*sll1456* PE=4 SV=1 |
| 90 | Citrate synthase OS=Synechocystis sp. (strain PCC 6803 / Kazusa) GN=*gltA* PE=3 SV=1 |
| 91 | Deoxycytidine triphosphate deaminase OS=Synechocystis sp. PCC 6803 GN=*dcd* PE=4 SV=1 |
| 92 | Glycogen operon protein GlgX OS=Synechocystis sp. PCC 6803 substr. PCC-N GN=*glgX* PE=4 SV=1 |
| 93 | Transcriptional repressor NrdR OS=Synechocystis sp. (strain PCC 6803 / Kazusa) GN=*nrdR* PE=3 SV=1 |
| 94 | Pyruvate kinase 2 OS=Synechocystis sp. (strain PCC 6803 / Kazusa) GN=*pyk2* PE=3 SV=1 |
| 95 | Carbon dioxide-concentrating mechanism protein CcmK homolog 2 OS=Synechocystis sp. (strain PCC 6803 / Kazusa) GN=*ccmK2* PE=1 SV=3 |
| 96 | 3-oxoacyl-[acyl-carrier-protein] reductase 1 OS=Synechocystis sp. (strain PCC 6803 / Kazusa) GN=*fabG1* PE=3 SV=1 |
| 97 | Uncharacterized protein OS=Synechocystis sp. PCC 6803 GN=BEST7613_5395 PE=4 SV=1 |
| 98 | Uncharacterized protein OS=Synechocystis sp. PCC 6803 GN=BEST7613_1916 PE=4 SV=1 |
| 99 | GDP-D-mannose dehydratase OS=Synechocystis sp. PCC 6803 GN=*rfbD* PE=4 SV=1 |
| 100 | Acetolactate synthase 3 catalytic subunit OS=Synechocystis sp. PCC 6803 substr. PCC-N GN=*ilvG* PE=3 SV=1 |
| 101 | 3-chlorobenzoate-3,4-dioxygenase OS=Synechocystis sp. PCC 6803 substr. PCC-N GN=*cbaB* PE=4 SV=1 |
| 102 | General secretion pathway protein E OS=Synechocystis sp. PCC 6803 GN=*gspE* PE=4 SV=1 |
| 103 | Transcriptional repressor NrdR OS=Synechocystis sp. PCC 6803 GN=*nrdR* PE=3 SV=1 |
| 104 | IMP dehydrogenase OS=Synechocystis sp. PCC 6803 GN=*guaB* PE=4 SV=1 |
| 105 | UDP-N-acetylmuramate--L-alanine ligase OS=Synechocystis sp. (strain PCC 6803 / Kazusa) GN=*murC* PE=3 SV=1 |
| 106 | Uncharacterized protein OS=Synechocystis sp. PCC 6803 GN=BEST7613_4900 PE=4 SV=1 |
| 107 | Uncharacterized protein OS=Synechocystis sp. PCC 6803 GN=BEST7613_4354 PE=4 SV=1 |
| 108 | Ferrochelatase OS=Synechocystis sp. (strain PCC 6803 / Kazusa) GN=*hemH* PE=3 SV=1 |
| 109 | Succinyl-CoA synthase subunit beta OS=Synechocystis sp. PCC 6803 GN=*sucD* PE=4 SV=1 |
| 110 | ABC transporter OS=Synechocystis sp. PCC 6803 GN=BEST7613_4903 PE=4 SV=1 |
| 111 | Putative OxPP cycle protein OpcA OS=Synechocystis sp. (strain PCC 6803 / Kazusa) GN=*opcA* PE=4 SV=1 |
| 112 | Protein RecA OS=Synechocystis sp. (strain PCC 6803 / Kazusa) GN=*recA* PE=3 SV=1 |
| 113 | OmpR subfamily protein Ycf27/Rre26 OS=Synechocystis sp. PCC 6803 substr. PCC-N GN=*rpaB* PE=4 SV=1 |
| 114 | Putative peroxiredoxin sll1621 OS=Synechocystis sp. (strain PCC 6803 / Kazusa) GN=*sll1621* PE=1 SV=1 |
| 115 | Phosphoglycerate kinase OS=Synechocystis sp. (strain PCC 6803 / Kazusa) GN=*pgk* PE=3 SV=2 |
| 116 | Light-dependent protochlorophyllide reductase OS=Synechocystis sp. (strain PCC 6803 / Kazusa) GN=*por* PE=3 SV=2 |
| 117 | Acyl carrier protein OS=Synechocystis sp. (strain PCC 6803 / Kazusa) GN=*acpP* PE=1 SV=2 |
| 118 | Adenylosuccinate synthetase OS=Synechocystis sp. (strain PCC 6803 / Kazusa) GN=*purA* PE=3 SV=2 |
| 119 | 16.6 kDa small heat shock protein, molecular chaperon OS=Synechocystis sp. PCC 6803 GN=*hsp17* PE=3 SV=1 |
| 120 | Uncharacterized protein OS=Synechocystis sp. PCC 6803 GN=BEST7613_6161 PE=4 SV=1 |
| 121 | Uncharacterized protein OS=Synechocystis sp. PCC 6803 GN=BEST7613_2166 PE=4 SV=1 |
| 122 | Uncharacterized protein OS=Synechocystis sp. PCC 6803 GN=BEST7613_1706 PE=4 SV=1 |
| 123 | Uncharacterized protein OS=Synechocystis sp. PCC 6803 GN=BEST7613_5897 PE=4 SV=1 |
| 124 | Uncharacterized protein OS=Synechocystis sp. PCC 6803 GN=MYO_3180 PE=4 SV=1 |
| 125 | Uncharacterized protein OS=Synechocystis sp. PCC 6803 substr. PCC-N GN=*sll1011* PE=4 SV=1 |
| 126 | Sensory transduction histidine kinase OS=Synechocystis sp. PCC 6803 GN=BEST7613_5402 PE=4 SV=1 |
| 127 | Uncharacterized protein OS=Synechocystis sp. PCC 6803 GN=BEST7613_4567 PE=4 SV=1 |
| 128 | Uroporphyrin-III synthase OS=Synechocystis sp. PCC 6803 substr. PCC-N GN=*hemD* PE=3 SV=1 |
| 129 | Photosystem I reaction center subunit III OS=Synechocystis sp. (strain PCC 6803 / Kazusa) GN=*psaF* PE=1 SV=1 |
| 130 | Uncharacterized protein OS=Synechocystis sp. PCC 6803 GN=BEST7613_2603 PE=4 SV=1 |
| 131 | tRNA (guanine-N(1)-)-methyltransferase OS=Synechocystis sp. (strain PCC 6803 / Kazusa) GN=*trmD* PE=3 SV=1 |
| 132 | Uncharacterized protein OS=Synechocystis sp. PCC 6803 GN=BEST7613_1577 PE=4 SV=1 |
| 133 | Branched-chain alpha-keto acid dehydrogenase E2 OS=Synechocystis sp. PCC 6803 GN=*odhB* PE=3 SV=1 |
| 134 | Coenzyme A biosynthesis bifunctional protein CoaBC OS=Synechocystis sp. (strain PCC 6803 / Kazusa) GN=*coaBC* PE=3 SV=1 |
| 135 | Uncharacterized WD repeat-containing protein sll0163 OS=Synechocystis sp. (strain PCC 6803 / Kazusa) GN=*sll0163* PE=4 SV=1 |
| 136 | Drug sensory protein A OS=Synechocystis sp. (strain PCC 6803 / Kazusa) GN=*dspA* PE=3 SV=2 |
| 137 | Uncharacterized protein OS=Synechocystis sp. PCC 6803 substr. PCC-N GN=*pleD* PE=4 SV=1 |
| 138 | LIM17 protein OS=Synechocystis sp. PCC 6803 GN=*invA* PE=4 SV=1 |
| 139 | Uncharacterized protein OS=Synechocystis sp. PCC 6803 substr. PCC-N GN=*slr0483* PE=4 SV=1 |
| 140 | Uncharacterized protein OS=Synechocystis sp. PCC 6803 substr. PCC-N GN=*sll1961* PE=4 SV=1 |
| 141 | Light-repressed protein A homolog OS=Synechocystis sp. (strain PCC 6803 / Kazusa) GN=*lrtA* PE=3 SV=1 |
| 142 | Lysozyme OS=Synechocystis sp. PCC 6803 substr. PCC-N GN=*slr0795* PE=3 SV=1 |
| 143 | Uncharacterized protein OS=Synechocystis sp. PCC 6803 substr. PCC-N GN=*slr0006* PE=4 SV=1 |
| 144 | Nitrate transport ATP-binding protein NrtC OS=Synechocystis sp. (strain PCC 6803 / Kazusa) GN=*nrtC* PE=3 SV=1 |
| 145 | Uncharacterized protein OS=Synechocystis sp. PCC 6803 GN=BEST7613_1696 PE=4 SV=1 |
| 146 | Photosystem I assembly protein Ycf4 OS=Synechocystis sp. (strain PCC 6803 / Kazusa) GN=*ycf4* PE=3 SV=1 |
| 147 | Probable inorganic polyphosphate/ATP-NAD kinase 1 OS=Synechocystis sp. (strain PCC 6803 / Kazusa) GN=*ppnK1* PE=3 SV=1 |
| 148 | Adenosylhomocysteinase OS=Synechocystis sp. (strain PCC 6803 / Kazusa) GN=*ahcY* PE=3 SV=1 |
| 149 | Universal stress protein Sll1654 OS=Synechocystis sp. (strain PCC 6803 / Kazusa) GN=*sll1654* PE=3 SV=1 |
| 150 | Arsenical resistance operon repressor OS=Synechocystis sp. PCC 6803 GN=*arsA* PE=4 SV=1 |
| 151 | Phosphoribosylformylglycinamidine synthase 1 OS=Synechocystis sp. (strain PCC 6803 / Kazusa) GN=*purQ* PE=3 SV=1 |
| 152 | Putative biopolymer transport protein ExbB-like 1 OS=Synechocystis sp. (strain PCC 6803 / Kazusa) GN=*sll0477* PE=3 SV=1 |
| 153 | Porphobilinogen deaminase OS=Synechocystis sp. (strain PCC 6803 / Kazusa) GN=*hemC* PE=3 SV=1 |
| 154 | Acetylglutamate kinase OS=Synechocystis sp. (strain PCC 6803 / Kazusa) GN=*argB* PE=3 SV=1 |
| 155 | Uncharacterized protein OS=Synechocystis sp. PCC 6803 substr. PCC-N GN=*slr1066* PE=4 SV=1 |
| 156 | dTDP-6-deoxy-L-mannose-dehydrogenase OS=Synechocystis sp. PCC 6803 substr. PCC-N GN=*rfbC* PE=4 SV=1 |
| 157 | Biosynthetic arginine decarboxylase 2 OS=Synechocystis sp. (strain PCC 6803 / Kazusa) GN=*speA2* PE=3 SV=1 |
| 158 | Uncharacterized protein sll0400 OS=Synechocystis sp. (strain PCC 6803 / Kazusa) GN=*sll0400* PE=3 SV=1 |
| 159 | Regulatory components of sensory transduction system OS=Synechocystis sp. PCC 6803 GN=BEST7613_2593 PE=4 SV=1 |
| 160 | Uncharacterized protein OS=Synechocystis sp. PCC 6803 GN=BEST7613_4515 PE=4 SV=1 |
| 161 | Uncharacterized monothiol glutaredoxin ycf64-like OS=Synechocystis sp. (strain PCC 6803 / Kazusa) GN=*slr1846* PE=3 SV=1 |
| 162 | Uncharacterized protein OS=Synechocystis sp. PCC 6803 GN=MYO_2130 PE=4 SV=1 |
| 163 | Transcription termination/antitermination protein NusG OS=Synechocystis sp. (strain PCC 6803 / Kazusa) GN=*nusG* PE=3 SV=1 |
| 164 | Phosphoribosylformylglycinamidine synthase 2 OS=Synechocystis sp. (strain PCC 6803 / Kazusa) GN=*purL* PE=3 SV=2 |
| 165 | 50S ribosomal protein L2 OS=Synechocystis sp. (strain PCC 6803 / Kazusa) GN=*rplB* PE=3 SV=1 |
| 166 | Chorismate synthase OS=Synechocystis sp. (strain PCC 6803 / Kazusa) GN=*aroC* PE=3 SV=2 |
| 167 | ABC transporter OS=Synechocystis sp. PCC 6803 GN=BEST7613_2590 PE=4 SV=1 |
| 168 | Phycobilisome 32.1 kDa linker polypeptide, phycocyanin-associated, rod 2 OS=Synechocystis sp. (strain PCC 6803 / Kazusa) GN=*cpcC2* PE=1 SV=1 |
| 169 | Naphthoate synthase OS=Synechocystis sp. PCC 6803 GN=*menB* PE=3 SV=1 |
| 170 | Photosystem I biogenesis protein BtpA OS=Synechocystis sp. (strain PCC 6803 / Kazusa) GN=*btpA* PE=3 SV=1 |
| 171 | ATP synthase gamma chain OS=Synechocystis sp. (strain PCC 6803 / Kazusa) GN=*atpG* PE=3 SV=1 |
| 172 | Sulfite reductase [ferredoxin] OS=Synechocystis sp. (strain PCC 6803 / Kazusa) GN=*sir* PE=1 SV=1 |
| 173 | Uncharacterized protein OS=Synechocystis sp. PCC 6803 GN=BEST7613_2510 PE=4 SV=1 |
| 174 | Ketol-acid reductoisomerase OS=Synechocystis sp. (strain PCC 6803 / Kazusa) GN=*ilvC* PE=1 SV=3 |
| 175 | Uncharacterized protein OS=Synechocystis sp. PCC 6803 GN=BEST7613_2675 PE=4 SV=1 |
| 176 | ATP synthase subunit delta OS=Synechocystis sp. (strain PCC 6803 / Kazusa) GN=*atpH* PE=3 SV=1 |
| 177 | Uncharacterized protein OS=Synechocystis sp. PCC 6803 GN=BEST7613_1691 PE=4 SV=1 |
| 178 | CTP synthase OS=Synechocystis sp. (strain PCC 6803 / Kazusa) GN=*pyrG* PE=3 SV=1 |
| 179 | Putative serine protease HhoA OS=Synechocystis sp. (strain PCC 6803 / Kazusa) GN=*hhoA* PE=1 SV=1 |
| 180 | Uncharacterized protein OS=Synechocystis sp. PCC 6803 GN=BEST7613_2061 PE=4 SV=1 |
| 181 | Urease subunit gamma OS=Synechocystis sp. (strain PCC 6803 / Kazusa) GN=*ureA* PE=3 SV=1 |
| 182 | Acetolactate synthase OS=Synechocystis sp. PCC 6803 GN=*ilvB* PE=3 SV=1 |
| 183 | HlyB family OS=Synechocystis sp. PCC 6803 GN=*hlyB* PE=4 SV=1 |
| 184 | Delta-aminolevulinic acid dehydratase OS=Synechocystis sp. (strain PCC 6803 / Kazusa) GN=*hemB* PE=1 SV=1 |
| 185 | Probable RuBisCO transcriptional regulator OS=Synechocystis sp. (strain PCC 6803 / Kazusa) GN=*rbcR* PE=3 SV=1 |

Table S2. MS/MS identification of CupAR (sll1736) and CupA (sll1734) in the fraction eluted by 150 mM imidazole from NiNTA column loaded with solubilized thylakoid membrane of the with CupA-His6 tag.

| CupA-his | PepCount | Unique Pepcount | CoverPercent | MW | PI | IdentifiedName |  |
| --- | --- | --- | --- | --- | --- | --- | --- |
|  | 45 | 12 | 37.35% | 50038.76 | 5.55 | GN=*Sll1734*(*CupA*) |  |
|  | 13 | 1 | 17.32% | 13773.11 | 5.69 | GN=*Sll1736*(*CupAR*) |  |

Table S3. Primers for this study

| Name | Sequence (5’-3’) | Description |
| --- | --- | --- |
| *cupAR*-Cm Up kpnI F | CGGGGTACCATTACCACCCTGGTACAAAATATT | Amplification of *ΔcupAR* gene (overlap, used for constructing vector) |
| *cupAR*-Cm UP BamH R | CGCGGATCCAGCCACCAGCGGAATACCAAAAA |  |
| *cupAR*-Cm Down PstI F | AAAACTGCAGGTGTTGCCCTAATGTATTCCAGCAA |  |
| *cupAR*-Cm Down HindIII R | CCCAAGCTTCCGGTGGACTTTGAACAACCCGGTA |  |
| *cupAR*-1F | CATTTGGCTAAGAATCGGCAATTTT |  |
| *cupAR*-1R | CGTCACAGGTATAGATGGCTCCTACCAACAGCATTACC |  |
| *cupAR*-2F | TAGGAGCCATCTATACCTGTGACGGAAGATCACTTC |  |
| *cupAR*-2R | ATTAGGGCAACACATACTGTTATCTGGCTTTTAGTAA |  |
| *cupAR*-3F | AGATAACAGTATGTGTTGCCCTAATGTATTCCAGCAA |  |
| *cupAR*-3R | GTTATCCCTCCCGGGGCGATCGTTG |  |
| *cupS*-F | ATGCCTAACATTGTTGAAATTGCGG | Amplification of *cupS and* *cupAR* gene  (used for identification) |
| *cupAR*-R | TCAGGACTCATCTAGTGCTTTAACT |  |
| YTH-*ndhD3*-F | AAGCAGGCTCCATGCTTAGCCTGCTCTTAAT | Amplification of *ndhD3-BD* gene (used for yeast two-hybrid test) |
| YTH-*ndhD3*-R | GAAAGCTGGGTTTTATGGCAAGGTCACCCCTT |  |
| YTH-*ndhF3*-F | AAGCAGGCTCCATGTTAGAAAGTTTAAGTCG | Amplification of *ndhF3-BD* gene (used for yeast two-hybrid test) |
| YTH-*ndhF3*-R | GAAAGCTGGGTTTTAAAATGCTTGGCCAAGCA |  |
| YTH-*cupA*-F | AAGCAGGCTCCATGACTACCCTGACCCCAAA | Amplification of *cupA-BD* gene (used for yeast two-hybrid test) |
| YTH-*cupA*-R | GAAAGCTGGGTTTTAACGATAACTATCGCTTT |  |
| YTH-*cupS*-F | AAGCAGGCTCCATGCCTAACATTGTTGAAAT | Amplification of *cupS-BD* gene (used for yeast two-hybrid test) |
| YTH-*cupS*-R | GAAAGCTGGGTTCTATCCCATTAAAATCACGT |  |
| YTH-*cupAR*-F | AAGCAGGCTCCGTGAACAACTTTTCCCCCAA | Amplification of *cupAR-AD* gene (used for yeast two-hybrid test) |
| YTH-*cupAR*-R | GAAAGCTGGGTTTCAGGACTCATCTAGTGCTT |  |
| YTH-*cupAR*-NM-F | AAGCAGGCTCCGTGAACAACTTTTCCCCC | Amplification of *cupAR-NM-AD* gene (used for yeast two-hybrid test) |
| YTH-*cupAR*-NM-R | GAAAGCTGGGTTTTACACCAATTTTCTCTTCAGC |  |
| *cupAR*-YFP Bam F | CGCGGATCCACGTGGTTGCCGGGAAGTTTAC | Amplification of *cupAR-YFP* gene (used for constructing vector) |
| *cupAR*-YFP Kpn R | CGGGGTACCGGACTCATCTAGTGCTTTAACTGAT |  |
| *cupA*-F | AAAGCCAACGCAGATAAAGCGATTC | Amplification of *cupA* gene (RT-PCR) |
| *cupA*-R | TCGGCATACAAAATACCATAGTGAA |  |
| *cupS*-F | TACGGTTTTCGCTCCCACGGATACG | Amplification of *cupS* gene (RT-PCR) |
| *cupS*-R | CGGGGATGATTACCGTGGCATTTTT |  |
| *cupAR*-F | AAGCAGGCTCCGTGAACAACTTTTCCCCCAA | Amplification of *cupAR* gene (RT-PCR) |
| *cupAR*-R | GAAAGCTGGGTTTCAGGACTCATCTAGTGCTT |  |
| *ndhF3*-F | ATTATTCTCAACAATGTGACCCAGG | Amplification of *ndhF3* gene (RT-PCR) |
| *ndhF3*-R | AGGATTGAGGTAAATAAAGGCTGAG |  |
| *ndhD3*-F | TGGATGTATTGAATGGTTTGATGAA | Amplification of *ndhD3* gene (RT-PCR) |
| *ndhD3*-R | CTGAACACCGAGGAAGAAAATAATG |  |
| *ndhD4*-F | GTACCGGATCCGTGGGTGGAATTTGCCCCCTATTTAG | Amplification of *ndhD4* gene (RT-PCR) |
| *ndhD4*-R | CAAGCTTGTCGACCGACACTTCCCCCAATTCCTTGACTTCA |  |
| *cupB*-F | GTACCGGATCCGTTAGCTTTTTTGATGGAAACCGCAG | Amplification of *cupB* gene (RT-PCR) |
| *cupB*-R | AGCTTGTCGACCGAATCGGCCATGGTTGAGGGTTGGTT |  |
| *sbtA*-F | GACGGACTTCGTGGGACAATTGCAG | Amplification of *sbtA* gene (RT-PCR) |
| *sbtA*-R | AGCCCAATGAAAAGCGGTATGCACA |  |
| *16S*-F | CGACTGCTAATACCCAATGTGC | Amplification of *16S* gene (RT-PCR) |
| *16S*-R | GTCCCTCAGTGTCAGTTTCAGC |  |
| *ndhR*-1F | AGATGACGACGACAAGGTACCTCTTGGACAGGGTCGCAATGC | Amplification of *ΔndhR* gene (one step cloning, used for constructing vector) |
| *ndhR*-1R | GCGTAACATCGTTGCTGCTCCGTCCTTGACGAAGATGGTGAT |  |
| *ndhR*-2F | ATCACCATCTTCGTCAAGGACGGAGCAGCAACGATGTTACGC |  |
| *ndhR*-2R | GTTAAAAATCAATCAAGGTGATTGAACGAATTGTTAGGTGGCG |  |
| *ndhR*-3F | CGCCACCTAACAATTCGTTCAATCACCTTGATTGATTTTTAAC |  |
| *ndhR*-3R | TGCGGCCGCAAGCTTGTCGACAGCAACAGAAAGTGCCTTAGTCG |  |


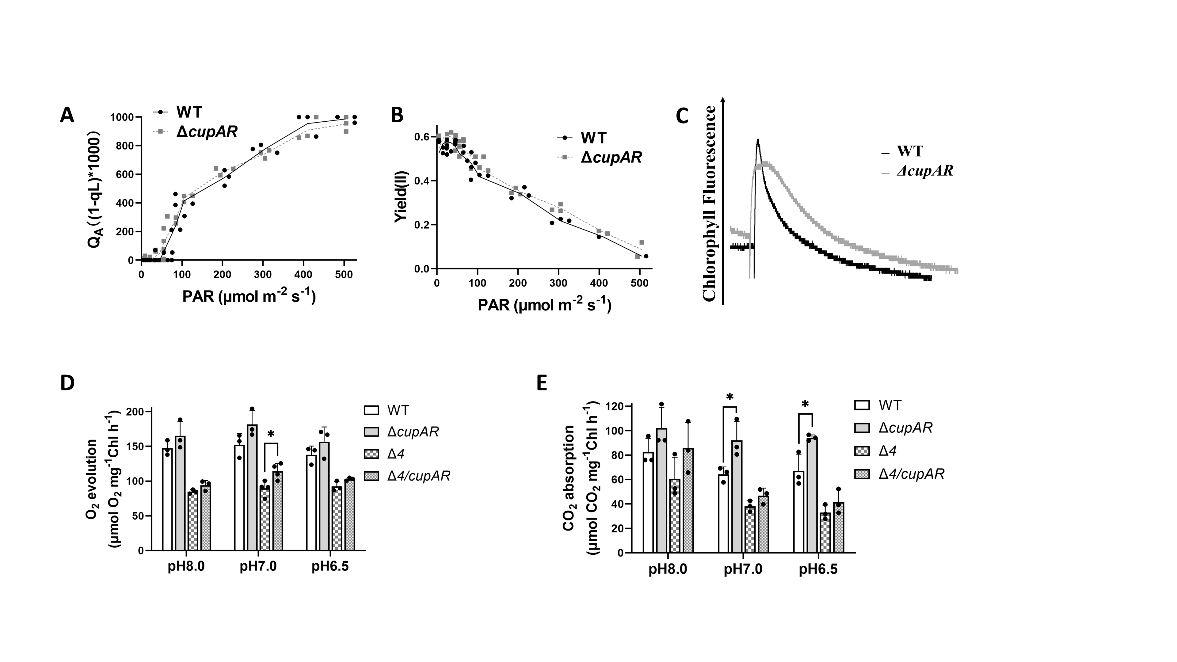


Figure S1. Comparison of photosynthetic capacities among WT and mutants. (A) Light curve of redox state of Q_A_ calculated as 1 – qL. (B) Light curve of the quantum yield of the PSII photochemistry [Yield (II)]. (C) The transient increase in chlorophyll fluorescence after termination of actinic light, which reflects NDH activity. (D) Photosynthetic oxygen evolution rates in WT and mutants. （E）CO_2_ uptake rate under 2% CO_2_ concentration at pH8.0, pH7.0 and pH6.5, respectively. Cells were grown in BG-11 under HC (2% CO_2_ (v/v) conditions. Significant differences (p < 0.05) are labeled with different letters on the column. Cells used for the experiments were at mid-logarithmic phase. Values represent the mean ±SE of three independent measurements.


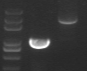


WT Δ*cupA*

ndhF3 ndhD3 cupA cupS cupAR

SpR

F

R

0.5—

0.75—

1—

2—

3—

5—

kbp

Figure S2. PCR segregation analysis of the *ΔcupA* using the *cupA*-up-F and *cupA*-down-R primers (Table S3) (top). Simplified scheme of the spectinomycin insertion mutant, *ΔcupA*. We inserted a spectinomycin-resistant (SpR) cassette into the *cupA* gene in the Syn6803 (bottom).

WT *cupACFP*
 1 2

kbp


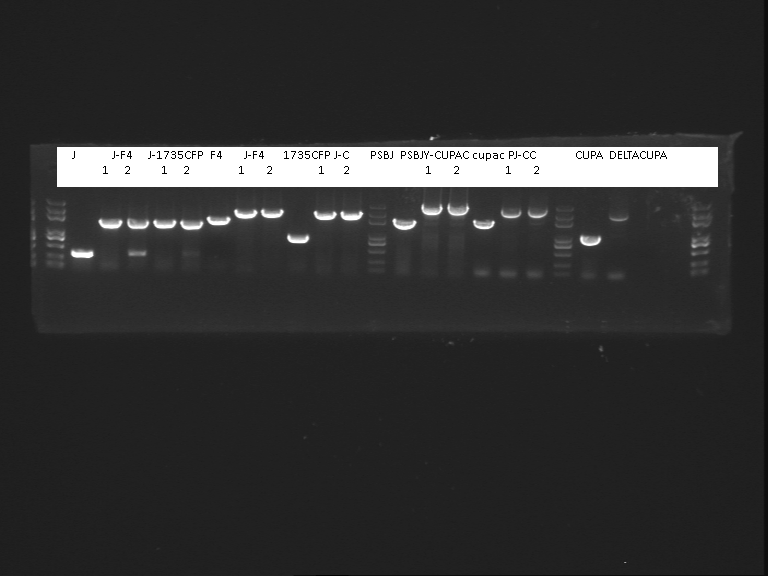


—5

—3

—2

—1

—0.75

—0.5

R

F

*ndhF3 ndhD3 cupA CFP KanR cupS cupAR*

Figure S3. PCR segregation analysis of the *cupA-CFP* using the *cupA*-up-F and *cups*-down-R primers (Table S3) (top). Simplified scheme of the *cupA*-*CFP*-kanamycin (kanR) replaced mutant, *cupA-CFP*. We replaced the *cupA* gene into a *cupA*-*CFP*-*kanR*-resistant cassette Syn6803 (bottom).


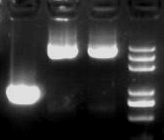


WT *cupARYFPHis*
 1 2

R

F

ndhF3 ndhD3 cupA cupS cupAR YFP-6His SpR

kbp

—5

—3

3.7kbp→

—2

—1

1kbp→

—0.5

—0.75

Figure S4. PCR segregation analysis of the *cupAR-YFP* using the *cupAR*-*YFP*-up-F and *cupAR-YFP*-down-R primers (Table S3) (top). Simplified scheme of *cupAR-YFP-6His*-spectinomycin (SpR) replaced mutant, *ΔcupAR-YFP-6His*. We replaced the *cupAR* gene into *cupAR-YFP-6His*-SpR cassette in the Syn6803 (bottom).

Reference

J.G. Williams, A.A. Szalay, Gene 24 (1983) 37.
